# Age-Dependent Ribosomal DNA Variations in Mice

**DOI:** 10.1128/MCB.00368-20

**Published:** 2020-10-26

**Authors:** Eriko Watada, Sihan Li, Yutaro Hori, Katsunori Fujiki, Katsuhiko Shirahige, Toshifumi Inada, Takehiko Kobayashi

**Affiliations:** a Institute for Quantitative Biosciences, University of Tokyo, Tokyo, Japan; b Department of Biological Sciences, University of Tokyo, Tokyo, Japan; c Collaborative Research Institute for Innovative Microbiology, University of Tokyo, Tokyo, Japan; d Graduate School of Pharmaceutical Sciences, Tohoku University, Sendai, Japan

**Keywords:** ribosomal RNA gene, rDNA copy number, DNA methylation, senescence, genome instability, mouse, mutation rate, yeast life span

## Abstract

The rRNA gene, which consists of tandem repetitive arrays (ribosomal DNA [rDNA] repeat), is one of the most unstable regions in the genome. The rDNA repeat in the budding yeast Saccharomyces cerevisiae is known to become unstable as the cell ages. However, it is unclear how the rDNA repeat changes in aging mammalian cells. Using quantitative single-cell analyses, we identified age-dependent alterations in rDNA copy number and levels of methylation in mice. The degree of methylation and copy number of rDNA from bone marrow cells of 2-year-old mice were increased by comparison to levels in 4-week-old mice in two mouse strains, BALB/cA and C57BL/6. Moreover, the level of pre-rRNA transcripts was reduced in older BALB/cA mice. We also identified many sequence variations in the rDNA. Among them, three mutations were unique to old mice, and two of them were found in the conserved region in budding yeast. We established yeast strains with the old-mouse-specific mutations and found that they shortened the life span of the cells. Our findings suggest that rDNA is also fragile in mammalian cells and that alterations within this region have a profound effect on cellular function.

## Abstract (precis)

The rRNA gene, which consists of tandem repetitive arrays (ribosomal DNA [rDNA] repeat), is one of the most unstable regions in the genome. The rDNA repeat in the budding yeast Saccharomyces cerevisiae is known to become unstable as the cell ages. However, it is unclear how the rDNA repeat changes in aging mammalian cells. Using quantitative single-cell analyses, we identified age-dependent alterations in rDNA copy number and levels of methylation in mice. The degree of methylation and copy number of rDNA from bone marrow cells of 2-year-old mice were increased by comparison to levels in 4-week-old mice in two mouse strains, BALB/cA and C57BL/6.

## INTRODUCTION

The genome, which comprises the complete set of genetic information in an organism, is sensitive to damage from environmental factors such as exposure to UV radiation. Damage to the genome is efficiently repaired by a highly organized repair system (1, 2). Nonetheless, some damage is not properly repaired, leading to mutations, which may include rearrangements such as deletions and amplifications. In addition, mutations can also arise from errors introduced during DNA replication. These mutations accumulate during successive cell divisions to induce cellular senescence. However, the underlying mechanism linking the accumulation of mutations to senescence is not well understood.

Damage to DNA tends to accumulate at fragile sites in the genome (3). In the budding yeast, Saccharomyces cerevisiae, the rRNA gene (ribosomal DNA [rDNA]) is known to be a fragile site that is related to cellular senescence (4). Eukaryotic rDNA is made up of repetitive tandem arrays, which in the case of the budding yeast comprises ∼150 rDNA copies located on chromosome XII. However, copies of these repeats are readily lost by homologous recombination. Because the cell requires a huge number of ribosomes, accounting for ∼60% of total cellular protein, a gene amplification system is needed to compensate for these losses. As a result, rDNA copy number frequently varies, leading to an unstable genomic region (for a review, see reference 5). In terms of rDNA gene amplification in budding yeast, the replication fork-blocking protein Fob1 works as a recombination inducer (6). Fob1 associates with the replication fork barrier (RFB) site, inhibiting the replication process and inducing a DNA double-strand break that triggers gene amplification/recombination (7–9).

Intriguingly, *fob1* mutants have a stable rDNA copy number, and life span is extended by ∼60% compared to that of the wild-type strain (10, 11). An important factor in suppressing changes in rDNA copy number is Sir2, an NAD^+^-dependent protein deacetylase that is conserved across all kingdoms of life. Interestingly, *sir2* mutants of S. cerevisiae display increased unequal sister chromatid recombination, and the rDNA copy number frequently changes (7, 12). Moreover, the life span of the *sir2* mutant is shortened to approximately half that of the wild-type strain (13, 14). Taken together, these observations suggest that rDNA instability (i.e., frequent copy number alteration) is related to senescence (15).

**FIG 1:**
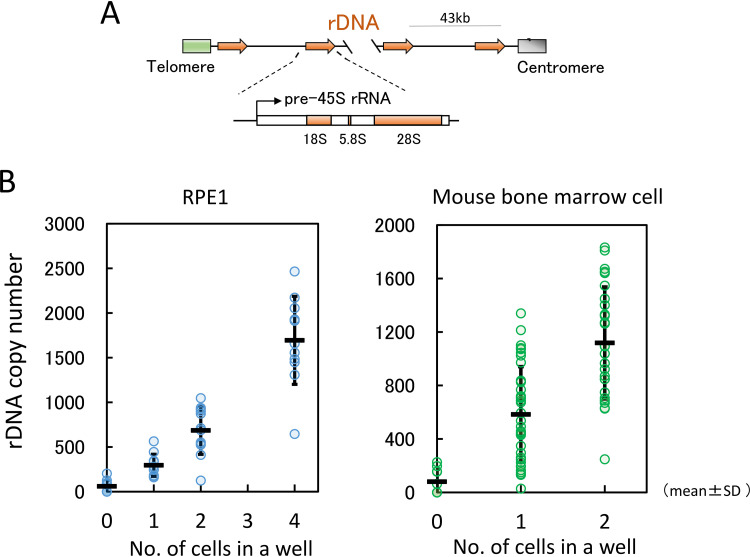
Accuracy of single cell analysis. (A) Structure of rDNA in mouse. One unit of rDNA is ∼43 kb, composed of the 45S pre-rRNA gene and intergenic spacer. 45S pre-rRNA is subsequently processed into three mature rRNAs (18S, 5.8S, and 28S). (B) rDNA copy numbers of one, two, and four cells were measured by qPCR. The rDNA copy number in RPE1 cells determined by digital PCR was used as the standard (see Materials and Methods). In all, 12 samples for RPE1 and 36 samples (represented by dots) for bone marrow cells were tested. The horizontal bars indicate the averages, and vertical bars indicate SDs.

In mammals, the rDNA structure is similar to that of yeast. However, the intergenic spacer sequence (IGS) in mammalian cells is larger than that in yeast and is an unstable region of the genome (Fig. 1A) (16). The connection between aging and rDNA has been suggested in several studies of tissues from dog, mouse, and human (17–21). Werner syndrome is a human premature aging disease. The rDNA of cells derived from patients with Werner syndrome display an increased level of noncanonical arrangements (22). In the hematopoietic stem cells of mice, replication stress accumulates in the rDNA, and cellular functional activity declines with age (23). However, there is still a paucity of observations on how rDNA changes during senescence.

Here, we compared the genomes of young and old mice and identified differences in rDNA stability, methylation, and transcription status. We also identified two mutations in rDNA that are specific to old mice. Moreover, these sequences are conserved in budding yeast rDNA. Interestingly, equivalent mutations in the budding yeast rDNA shortened the yeast chronological life span. These findings suggest that the rDNA is also fragile in a mammalian cell and that mutation of these sites affects cellular function.

## RESULTS

### rDNA copy number is increased in older mice.

Because the rDNA copy number readily changes, each cell may have a different copy number. Therefore, we initially measured the rDNA copy number in a single cell by quantitative real-time PCR (RT-qPCR). With this strategy, we determined the rDNA copy number of RPE1 (human retinal pigment epithelial) cells to obtain a standard curve by droplet digital PCR (ddPCR; Bio-Rad). In brief, a fixed amount of RPE1 DNA was digested into small fragments, diluted, and fractionated into droplets. The dilution factor ensured that each droplet contained just one DNA fragment. Each droplet was then subjected to PCR, and the number of positive droplets with an rDNA fragment was counted. The ratio of the number of positive to negative droplets gives the absolute copy number of rDNA. Using this method, RPE1 cells were found to have 330 rDNA copies (see Materials and Methods for details). The RPE1 DNA was then used as a control in determining the mouse rDNA copy number in a single cell by qPCR. Initially, we ensured the accuracy of the assay using one and two bone marrow cells and one, two, and four RPE1 cells to measure the rDNA copy number by qPCR. As anticipated, the rDNA copy number increased linearly with cell number (Fig. 1B).

**FIG 2:**
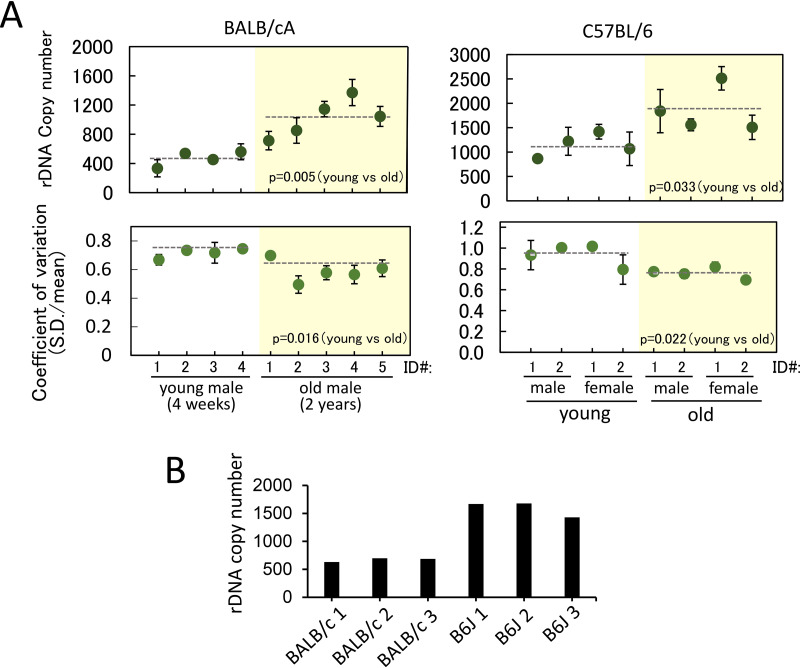
rDNA copy number and coefficient of variation in individual cells. (A) rDNA copy numbers were measured in young (4-week-old) and old (2-year-old) mice. rDNA copy number in single cells were measured by qPCR and plotted (top). The copy number was determined using RPE1 as a standard (see Materials and Methods). The *x* axis shows the identification (ID) numbers of individual mice used to isolate the bone marrow cells. The dotted lines represent the average values of the mice. The green dots represent the average of two independent qPCR experiments from each mouse, and the error bars indicate the range. Plots of coefficient of variation (SD divided by the average) for each mouse are shown (bottom). (B) Estimated rDNA copy number based on a whole-genome sequencing database. rDNA copy numbers of BALB/cA (BALB/c 1 to 3) and C57BL/6 (B6J 1 to 3) mice were estimated by reanalysis of a publicly available whole-genome sequencing database. rDNA copy number estimation based on whole-genome sequencing data was performed as follows. Fastq files obtained from the NCBI Sequence Read Archive (SRA; accession numbers PRJNA41995 and PRJNA386034) were mapped against mouse whole-genome and rDNA sequences using Bowtie 2, and the fraction of rDNA reads among all mapped reads was used to calculate the copy numbers.

Bone marrow cells were isolated from young (4-week-old) and old (2-year-old) BALB/cA and C57BL/6 mice. Specifically, four young and five old BALB/cA mice (males) and four young (two males and two females) and four old (two males and two females) C57BL/6 mice were tested. The cells were separated into a 96-well plate using a fluorescence-activated cell sorting (FACS) machine and subjected to qPCR to determine the rDNA copy number. The results are shown in Fig. 2A. The average rDNA copy number per cell (dotted lines) was significantly different in these two strains. Specifically, the average rDNA copy numbers per cell in young BALB/cA and C57BL/6 mice were 471 and 1,025, respectively. Thus, compared to BALB/cA mice, C57BL/6 mice were found to have more than double the number of rDNA copies per cell. The corresponding ratio of rDNA copy number for C57BL/6 to BALB/cA (1,025/471) mice was 2.18. To confirm this difference, we also estimated rDNA copy number using publicly available whole-genome sequencing data in NCBI. As shown in Fig. 2B, data for three mice from each strain were analyzed. The average rDNA copy numbers per cell were determined as 642 for BALB/cA mice and 1,412 for C57BL/6 mice. The corresponding ratio of rDNA copy number for C57BL/6 to BALB/cA (1,412/642) mice was 2.20. Based on these observations, we concluded that the difference in rDNA copy numbers between the two strains in our single-cell analysis was reasonable.

Next, the aging effect on rDNA copy number was investigated. For both mouse strains, the average rDNA copy number increased in the older mice. We also calculated the coefficient of variation (standard deviation [SD]/mean) in individual cells, which indicates the rate of copy number variation in each mouse cell normalized by the average value. The values obtained for the old mice were smaller than those for the young mice (see Discussion). These findings indicated that the rDNA copy number increased in the cells of most old mice while the copy number variation decreased.

**FIG 3:**
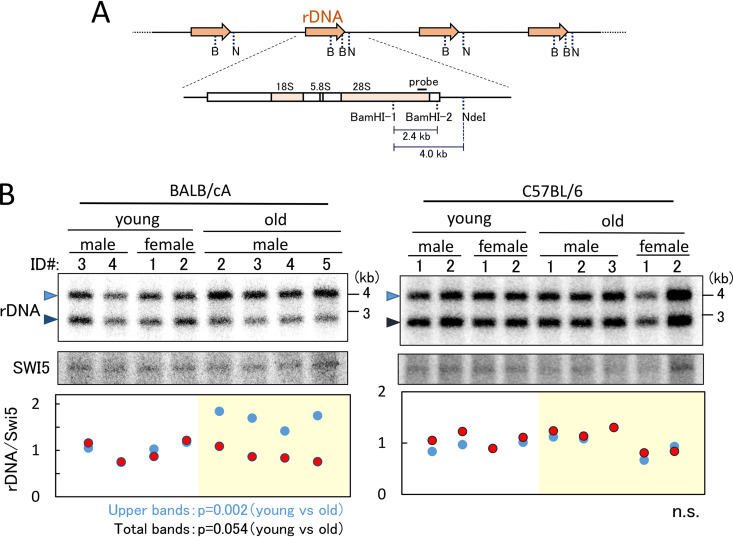
Detection of relative rDNA copy number in old and young mice. (A) The position of the probe for the Southern blot analysis shown in panels B and C and recognition sites for BamHI (B) and NdeI (N) are shown. (B and C) Detection of relative rDNA copy number. Southern analysis for rDNA copy number is shown at the top. DNA was digested with BamHI and NdeI. Upper bands (4 kb) come from rDNA units without the BamHI-2 site, and lower bands (2.4 kb) are from rDNA units with the BamHI-2 site. The SWI5 gene was used as a loading control (middle). The single-copy gene SWI5 was detected on the same filter used for the experiment shown in the upper panel. Relative rDNA copy numbers were determined (bottom). The band intensities of rDNA were normalized by those of SWI5, and the values are relative to the average of rDNA values in the four young mice. The blue dots show the results from the upper-band intensities of rDNA, and the red dots are the results from the lower bands. Individual mice that were used to isolate the bone marrow cells are identified by number (ID) (Fig. 2). *P* values are shown at the bottom of the panel. n.s., not significant.

We also tested the copy number alteration in old mice by Southern blotting. In this assay, DNA was isolated from mouse bone marrow cells and double digested with BamHI/NdeI restriction endonucleases before being subjected to agarose gel electrophoresis (Fig. 3). The probe for the Southern blot was designed to recognize the 28S rRNA gene in the 4-kb BamHI-NdeI-restricted fragment. However, some of the rDNA copies had a second BamHI site (BamHI-2) in the 4-kb fragment (Fig. 3A), resulting in the detection of two bands (Fig. 3B, top). For BALB/cA mice, the upper bands (4 kb) appeared stronger than the lower bands in the old mice, suggesting a relative loss of BamHI-2 sites within the rDNA. To normalize the results, a single-copy gene (SWI5) was also detected using a specific probe (Fig. 3B, middle). The intensities of the bands were measured, and the values were plotted (Fig. 3B, bottom). This analysis showed that the intensity of the 4-kb BamHI-NdeI fragment for BALB/cA mice increased relative to the levels for the other fragments. Taken together, the data showed that the rDNA copy number tended to increase with age although the difference was not as marked as in the qPCR analysis (see Discussion).

### rDNA transcription levels are decreased in older mice.

**FIG 4:**
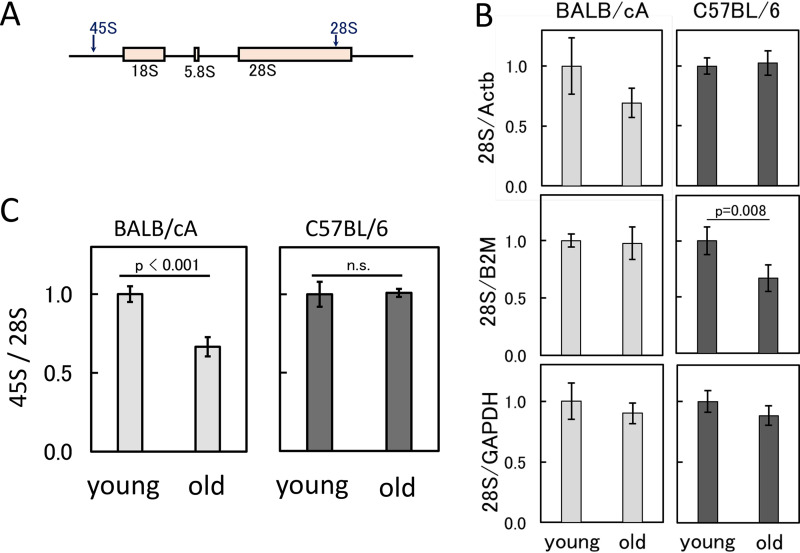
rDNA transcripts in old and young bone marrow cells. (A) Positions of the primer sets for qPCR to measure rDNA transcripts (pre-45S and 28S rRNA). (B) Amount of 28S rRNA normalized by transcripts of the three housekeeping genes (GAPDH, B2M, and Actb genes). The values are the average of four independent experiments, and the error bars indicate SDs. The values are relative to those of young cells. *P* values are shown if significant (*P* < 0.05). (C) Ratio of the pre-45S rRNA to 28S rRNA. The values are the average of four independent experiments, and the error bars indicate SDs. The values are relative to those of young cells. n.s., not significant.

The previous qPCR and Southern analysis showed that the copy number of the rDNA tended to increase in older mice. We therefore speculated that the increased rDNA copy number might result in an elevated level of rDNA transcripts (rRNAs). To test this hypothesis, RNA was isolated using cells derived from young and old mice, and the level of 28S rRNA was measured by RT-qPCR. The values were normalized against levels of the transcripts of three housekeeping genes, the Actb (actin, beta), B2M (beta-2 microglobulin), and glyceraldehyde-3-phosphate dehydrogenase (GAPDH) genes. The results are shown in Fig. 4B. Although there was a tendency for the cells of young mice to have more 28S rRNA, the difference was not significant except for the results normalized against the level of B2M. It is possible that the housekeeping genes are also affected by age. In addition, most of the 28S rRNAs are thought to be included in the ribosomes that abundantly accumulate in the cell. Therefore, we measured newly synthesized premature 45S rRNA using a probe that recognizes the promoter region and then calculated the ratio of premature to mature rRNA. As shown in Fig. 4C, in BALB/cA mice, the ratio of newly synthesized to mature rRNA was reduced in the old mice. However, this difference was not as obvious in the C57BL/6 mice.

**FIG 5:**
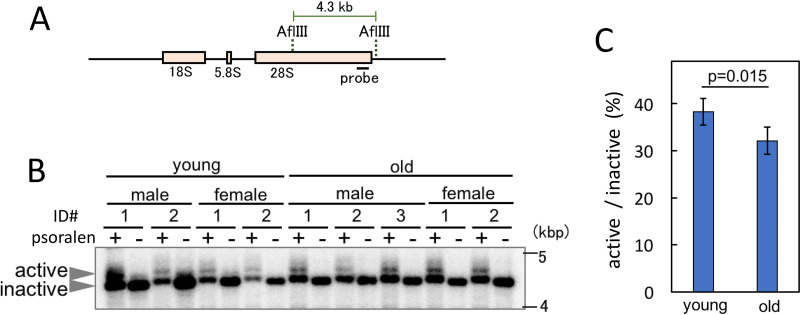
Ratio of active to inactive rDNA in old and young cells. Psoralen cross-linked rDNA was digested with AflIII, and band retardation was assessed after electrophoresis. (A) Recognition sites of AflIII and position of the probe for the experiment shown in panel B. (B) Southern blot analysis to detect the psoralen cross-linked rDNA by mobility retardation in young and old mice. Transcription-active rDNA efficiently intercalates psoralen, which retards migration during gel electrophoresis. Mouse identification (ID) numbers are given (Fig. 3B). (C) Ratio of copy numbers of active to inactive rDNA. Band intensities from the experiment shown in panel B were measured, and the ratio of active to inactive rDNA was calculated. The error bars represent SDs.

Transcription inactivation of the rRNA gene in C57BL/6 mice was confirmed using the psoralen cross-linking method (24). Psoralen intercalates into nonnucleosomal rDNA copies that are actively transcribed more efficiently than those that are transcriptionally inactive. Therefore, using this method, we can estimate the proportion of active rDNA copies. Cells were treated with psoralen and UV cross-linked, and the DNA was isolated. After digestion with AflIII, the DNA was subjected to agarose gel electrophoresis. The results are shown in Fig. 5. The upper and lower bands correspond to transcribed (active) and nontranscribed (inactive) rDNA copies, respectively (Fig. 5B). Band intensities were measured, and the values were plotted (Fig. 5C). The ratio of active to nonactive rDNA was less in the old cells than in the young cells. These findings suggest that rDNA transcription is reduced in the older mice.

### rDNA is more highly methylated in the older mice.

**FIG 6:**
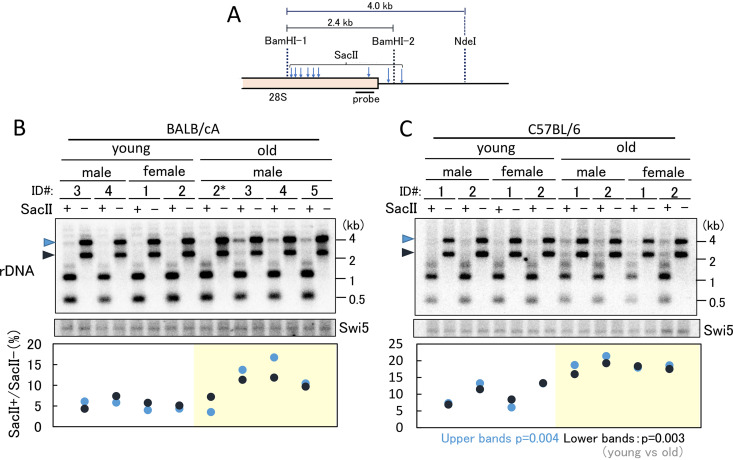
rDNA methylation in old and young bone marrow cells. rDNA methylation was detected by digestion with the methylation-sensitive enzyme SacII. (A) Position of the probe for Southern blot analysis in panels B and C and recognition sites for BamHI and SacII. (B and C) Ratio of copy numbers of methylated rDNA in young and old mice. Southern blot analysis was used to detect the ratio in undigested bands by SacII (top). The positions of undigested bands are indicated by arrowheads on the left-hand side of the panels. The SWI5 gene served as a loading control (middle). A single-copy gene, SWI5, was detected on the same filter used for the experiment shown in the upper panel. Analysis of rDNA that failed to digest with SacII was performed (bottom). The signal intensities of the undigested rDNA (SacII^+^) and nondigested (SacII^−^) bands were measured, and the ratios were plotted. The black and blue dots show the results corresponding to the bands indicated by the black and blue arrowheads, respectively, in the top panel. Identification (ID) numbers of individual mice that were used to isolate the bone marrow cells are given (Fig. 3). *P* values were calculated from the average of the values for young and old mice.

Transcription is known to be affected by DNA methylation (25). Recently, it was reported that the methylation rate of rDNA increases in an age-dependent manner in both mouse and human (26). Therefore, we speculated that increased methylation of rDNA might reduce the transcription level in older mice. To test this hypothesis, DNA from the old and young mice was digested using a methylation-sensitive enzyme, SacII, and the restriction pattern was analyzed (27). As shown in Fig. 6B, in the absence of SacII, two bands (4.0 and 2.4 kb, highlighted by arrowheads) were observed after BamHI-NdeI digestion (compare with Fig. 3B). However, after SacII digestion, most of these bands disappeared in the young mice. In contrast, the same analysis of DNA from old mice showed that faint bands were still detectable (Fig. 6B). The signal intensities of undigested and digested bands were measured, and the ratios were calculated. As a loading control, a single gene, the SWI5 gene, was also detected. The values of signal intensity were then plotted (Fig. 6B, lower panel). The ratios of methylation in the old mice were increased except for one mouse (mouse 2*). The same assay was performed in the C57BL/6 mouse strain, and similar results were obtained (Fig. 6C). These results confirmed that rDNA in the old mice is more methylated than that in the young mice. Taken together, our findings suggest that DNA methylation caused the reduced level of transcription of rDNA.

### There is sequence variation in rDNA of young and old mice.

**FIG 7:**
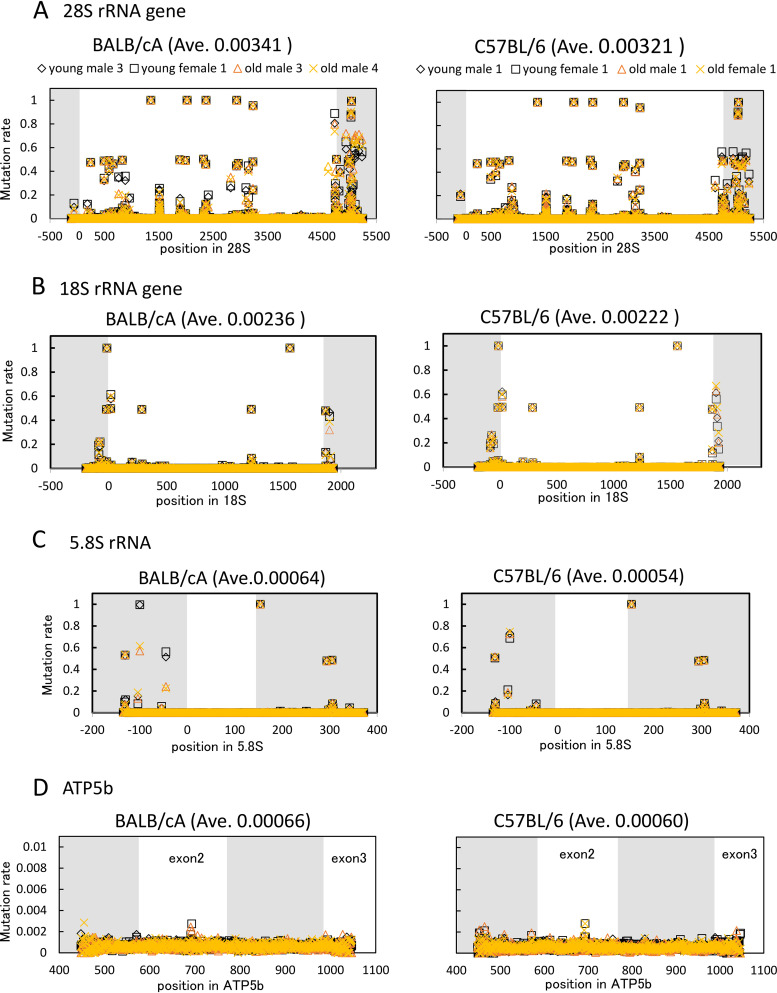
rDNA sequence variation in young and old mice. The rDNA sequences from two old and two young mice were determined and compared to the reference sequence. Mutation rates were then calculated at each nucleotide position for the indicated genes. Noncoding regions are shown in gray. The mutation rate is the difference in the sequence from the reference sequence. Ave, average mutation rate.

Finally, we determined the rDNA sequence in the young and old mice. Bone marrow cells, including hematopoietic stem cells that produce leukocytes, erythrocytes, and platelets, are known to divide frequently. Thus, we speculated that mutations in the older mouse cells would accumulate and affect the function of the ribosome, causing aging phenomena, such as slow growth and reduced viability. DNA from young and old mice was isolated, and the 18S, 5.8S, and 28S genes were PCR amplified for analysis by deep sequencing. All of the reads were aligned and compared with the mouse reference sequences (28) to identify mutation sites. The results are shown in Fig. 7A to C. The mutation rate is the ratio of mutations identified in the sequences to the total number of reads. Thus, a mutation rate of 1 (100%) means that the sequence is different from the reference sequence. If the mutation rate is 0.5 (50%), half of the rDNA copies display a variation at that site. As a control, we also analyzed a housekeeping gene, ATP5b (ATP synthase gene) (Fig. 7D).

**FIG 8:**
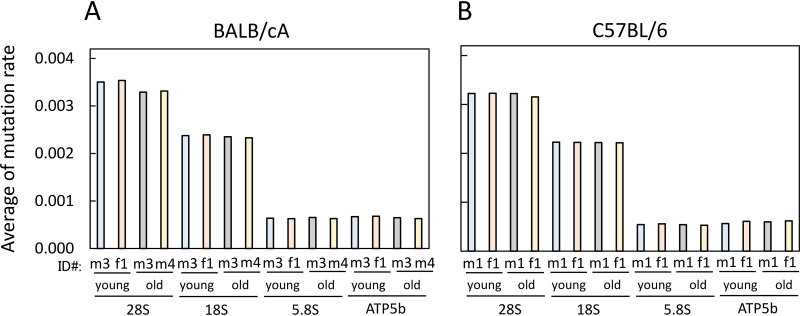
Mutation rates in rDNA of young and old mice. Average mutation rates of rDNA were calculated for the indicated BALB/cA (A) and C57BL/6 (B) mice. Mice are identified by number and sex (m, male; f, female). The same mice were used as for the experiment shown in Fig. 7. Mutation rates of >0.9 were not used because they are different from those of the reference sequences in nature.

As shown in Fig. 7, the overlapping black and yellow marks indicate that the mutation rates in the young and old mouse cells were similar. In conclusion, the average mutation rates in both young and old mouse cells were comparable (Fig. 8). Thus, any age-dependent alteration of rDNA sequence was not immediately apparent. Nonetheless, the average mutation rate of 28S rDNA (BALB/cA mice, 0.00341; C57BL/6 mice, 0.00321) was higher than that of 18S rDNA (BALB/cA mice, 0.00236; C57BL/6 mice, 0.00222) and much higher than the rates for the ATP5b and 5.8S genes (0.00054 to 0.00066). Indeed, sequence variation among copies of 28S rDNA has been reported previously (29). All of the high rate variations in 28S and 18S rDNAs were found in DNA from both young and old mice (Fig. 7).

**FIG 9:**
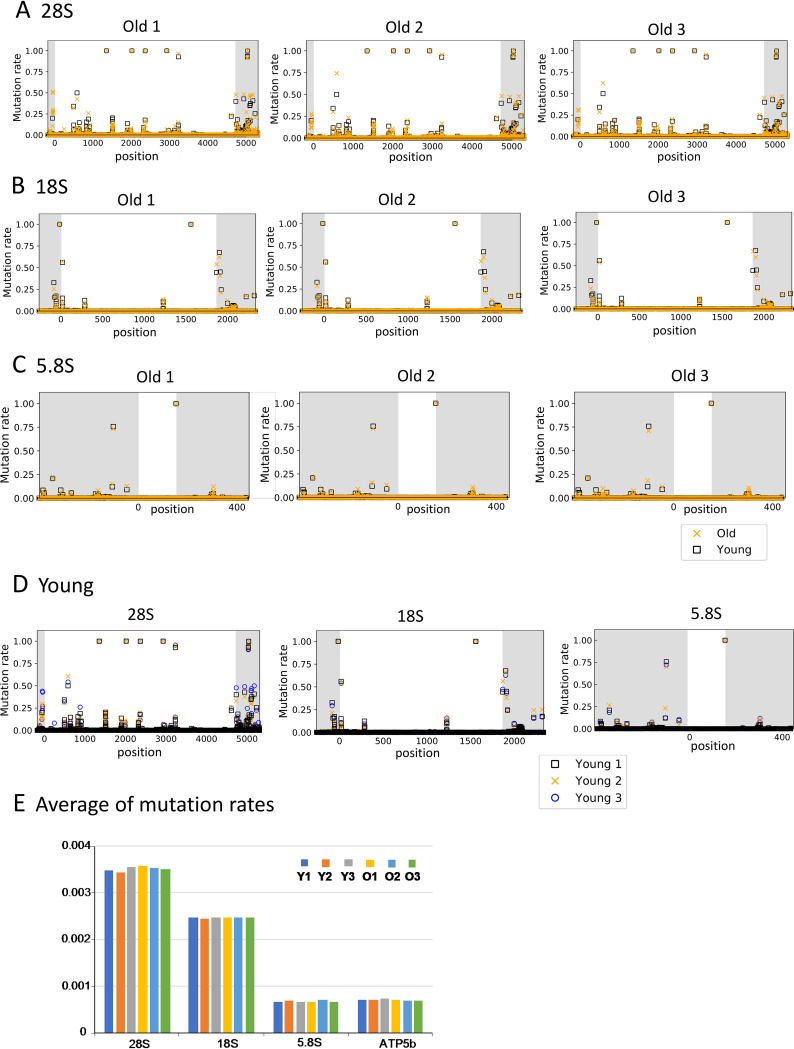
rDNA sequence variation in young and old C57BL/6 mice. rDNA sequences from three old (old 1 to old 3) and three young (young 1 to young 3) C57BL/6 mice were determined and compared to the reference sequence. The mutation rate at each nucleotide position was calculated as described in the legend to Fig. 7. (A to C) Mutations rates in old mice 1 to 3 were compared to the rate in young mouse 1 for the indicated genes. (D) Mutation rates in three young mice. (E) Average mutation rates. Noncoding regions are shown in gray. The mutation rate is based on differences from the reference sequence (as described in the legend to Fig. 8).

For the purpose of identifying old-mouse-specific mutations, we searched for variations with a mutation rate of >0.0028 (0.28%), which was equivalent to the maximum value for the control gene (ATP5b). The threshold value is the maximum apparent artificial mutation rate caused by PCR amplification or other errors. Within this range, we identified three old-mouse-specific mutations in old mice of the BALB/cA strain (Table 1). In contrast, no old-mouse-specific mutations were identified in the C57BL/6 strain. Indeed, no old-mouse-specific variations were found after the number of mice that were sequenced was increased (Fig. 9A to C). The average mutation rates in cells of both young (8-week-old) and old (100-week-old) mice were also comparable (Fig. 9E).

**TABLE 1:** The position and mutation rate of old-mouse-specific mutations in BALB/cA mice

Position of mouse 28S	Mutation rate in:						Difference in avg rate^*a*^	Position of yeast 25S
	Young mice			Old mice				
	Male 3	Female 1	Avg	Male 3	Male 4	Avg		
4614	0.000	0.001	0.001	0.441	0.396	0.419	0.418	3295
3291	0.001	0.001	0.001	0.039	0.032	0.035	0.034	2131
3094^*b*^	0.036	0.038	0.037	0.009	0.007	0.008	−0.029	

a Calculated as the difference between the averages for old mice and young mice.

b Position 3094 is not conserved in yeast rDNA.

Accuracy of the sequencing data was verified by analyzing variation of the BamHI recognition sequence that was detected (Fig. 3 and 6). The anticipated variation in the sequencing data corresponding to the BamHI site (GGATCC) in both mouse strains was observed together with the changes seen in the old BALB/cA mice (0.25 to 0.685) (Table 2). Thus, the sequencing data correlate with the Southern analysis in which the intensities of the upper bands increased in the old BALB/cA mice (Fig. 3B).

**TABLE 2:** Sequence variation at BamHI recognition sequences in young and old mice^*a*^

Position in 28S	Mutation rate^*b*^ in:							
	BALB/cA mice				C57BL/6 mice			
	Young (avg, 0.25)		Old (avg, 0.685)		Young (avg, 0.44)		Old (avg, 0.38)	
	Male 3	Female 1	Male 3	Male 4	Male 1	Female 1	Male 1	Female 1
G5050	0.00	0.00	0.00	0.00	0.00	0.00	0.00	0.00
G5051	0.00	0.00	0.00	0.00	0.00	0.00	0.00	0.00
A5052	**0.23**	**0.27**	**0.35**	**0.32**	**0.22**	**0.25**	**0.21**	**0.21**
T5053	**0.00**	**0.00**	**0.19**	**0.16**	**0.09**	**0.11**	**0.09**	**0.08**
C5054	**0.00**	**0.00**	**0.19**	**0.16**	**0.08**	**0.11**	**0.09**	**0.08**
C5055	0.00	0.00	0.00	0.00	0.00	0.00	0.00	0.00

a The sequences of the original and mutated BamHI sites are 5′-GGATCC-3′ and 5′-GGGGTC-3′, respectively (mutated sequence underlined). avg, average sum of the mutation rates.

b The mutation rates of mutated sequences are in boldface.

### The old-mouse-specific mutations of rDNA affect yeast life span.

**FIG 10:**
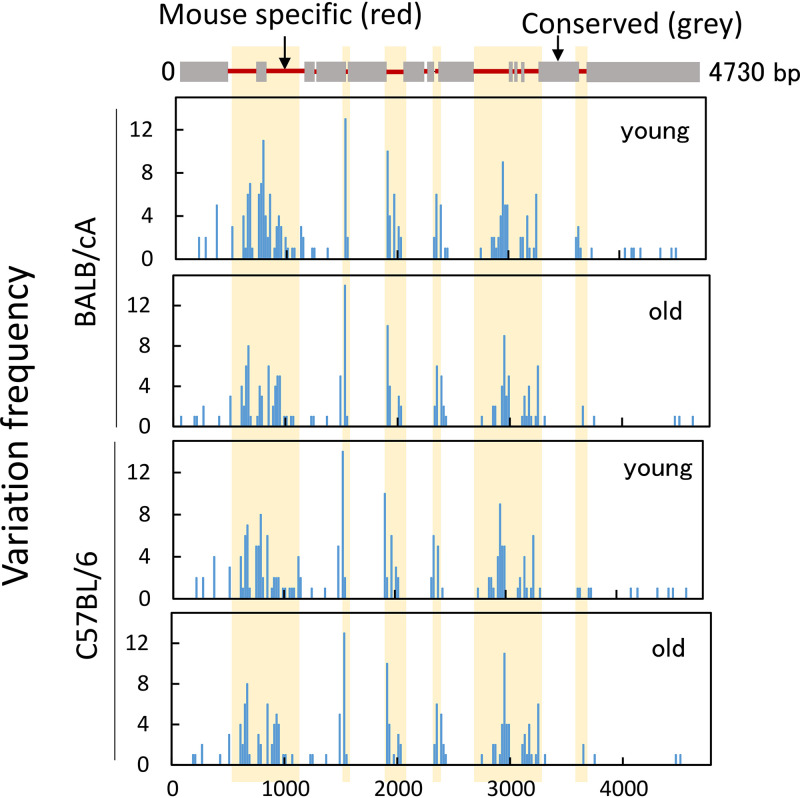
Hot spot of variation in the 28S rDNA. Mapping of hot spots of variation in the mouse 28S gene. The alignment of the mouse 28S and budding yeast 25S rRNA genes is shown (top). Variation frequency in the 28S rRNA gene was determined (bottom). The mutation rates were summed (variations with a mutation rate of >0.0028 were included) (Fig. 7A) every 20 bp and then plotted. Data from two mice were used in each graph. The yellow boxes represent mouse-specific nonconserved regions.

To analyze the relationship between rDNA variation and function, we summed the mutation rates in 20-bp windows and plotted the values (Fig. 10). In the graph, several variations, or hot spots, were identified over the 28S rDNA. Interestingly, most of the hot spots (highlighted in yellow) were located in the nonconserved regions between mouse and budding yeast rDNA (red line, top). These observations suggest that most of the variations are present in the nonfunctional region of the 28S rRNA gene.

**FIG 11:**
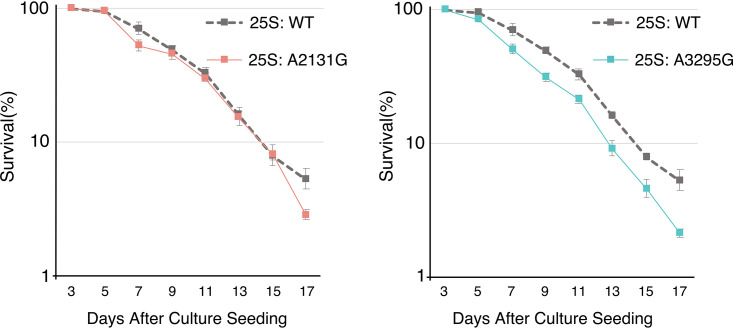
Chronological life span in budding yeast with mutated rDNA. Two old-mouse-specific mutations in the 28S rRNA gene were introduced into the budding yeast 25S rRNA gene, and the chronological life spans of the yeast were measured for the strains with A2131G and A3295G mutations in the 25S rRNA gene, as indicated. The values are the average of nine experiments. WT, wild type.

We also mapped the positions of the three old-mouse-specific mutations identified in the BALB/cA mice to yeast 25S rDNA. Interestingly, two sites (positions 3291 and 4614) were plotted in the conserved region between mouse and yeast, suggesting that they might be located in the functional domains in the rRNA. One approach to study the consequence of these mutations is to examine their impact in yeast. Thus, we generated budding yeast strains carrying the corresponding mutations in the 25S rDNA. For specific expression of the mutated rDNA, we used a yeast strain without rDNA in the chromosome (*rdn*ΔΔ strain) (30). The strain initially carried a helper rDNA plasmid, which was then shuffled with plasmids containing mutations in the 25S region. The plasmid-borne mutated rDNA thus became the sole source of rRNA. Strains with either plasmid-derived wild-type rDNA or A2131G (mouse A3291G) or A3295G (mouse A4614G) mutated rDNA showed comparable levels of cell growth in both solid and liquid media. To test the relationship between these mutations and senescence, we measured the chronological life span by calculating survival rates every 2 days after the cells entered the stationary phase. As shown in Fig. 11, one of the mutations (A3295G) lowered the proportion of surviving cells at all time points from day 5 onwards, indicating a shortened chronological life span. In contrast, another mutant (A2131G) showed survival rates similar to those of the wild-type yeast until day 15, but then the rate dropped on day 17. These observations suggest that although both mutations identified in the old mouse rDNA did not affect cell growth in yeast, they may be harmful during chronological aging, particularly the mutation A3295G (mouse A4614G).

## DISCUSSION

The rDNA has the following unique features that make it possible to monitor age-dependent alterations in the genome. First, because rDNA represents a highly repetitive and recombinogenic region, it is easy to assess instability by monitoring alterations in copy number (31–33). Second, as approximately half of the rDNA copies are not transcribed (24, 34), these repetitive nontranscribed regions are targets for both methylation (35) and mutation (36). Indeed, our analyses detected alterations in the copy number and methylation level in old mice, as well as putative old-mouse-specific mutations.

In terms of the rDNA copy number alteration observed in old mice, the results from literature reports are contradictory (18). Copy number alteration itself is commonly observed by many researchers; but in some reports the copy number goes up, and in others it goes down. Moreover, copy number alteration has also been observed in tissues (18). Some of these discrepancies may arise from problems related to hybridization during Southern blot analysis. The repetitive nature of the DNA combined with the high level of bound proteins from the nucleolus may affect the detection efficiency. Indeed, our results showed that although the rDNA copy number in old mice increased, as detected via single-cell analysis by qPCR, this increase was not obvious by Southern blotting in either of the two mouse strains (Fig. 2 and 3). For budding yeast, rDNA copy numbers in the old cells dramatically increase (∼10 times) as extrachromosomal rDNA circles (ERCs), and their presence is a big burden on the cells because ERCs consume factors that are required for chromosome maintenance (37, 38). Therefore, the copious amount of ERCs is thought to be a passive accelerator of cellular aging. In the case of mammals, this age-dependent increase in rDNA copies is not as dramatic (<2-fold) (Fig. 2). As such, the extra rDNA copies in mammals may not in themselves reduce life span.

In terms of genome instability, it may be possible to connect age-dependent changes in rDNA to the aging process. To address this issue, we previously established a strain of S. cerevisiae with reduced replication initiation activity only in the rDNA (39). Because ERCs cannot replicate, there is no ERC accumulation. However, the life span of the strain was shortened, and rDNA stability was reduced in the strain. We speculated that extended travel of DNA polymerase, due to reduced replication initiation, induces DNA replication stress, such as fork arrest and damage, leading to genome instability. These findings suggested that rDNA instability and/or damage itself is an aging signal that shortens life span (5). From this viewpoint, yeast and mammalian rDNAs may play similar roles in terms of aging by acting as large fragile sites for disseminating an aging signal (5). Indeed, the replication fork-blocking activity that causes rDNA recombination in the budding yeast is also present in mammalian rDNA (for a review, see references 40 and 41). A similar fork arrest induces rDNA instability to promote senescence by distributing the aging signal. Further studies are required to investigate this hypothesis.

In the single-cell analysis, we found that the copy number of rDNA increased, but variation decreased in older cells. As far as we are aware, there is no previous report showing alteration of rDNA copy number at the single-cell level. One possible reason to explain the reduced-variation phenotype in the older cells is that the number of stem cells for bone marrow decreases with age. Indeed, it is known that the number of the hematopoietic stem cells in the bone marrow gradually decreases during the process of aging (42). As bone marrow cells are produced from the stem cells, the variation in rDNA copy numbers is reduced.

The relationship between rDNA methylation and senescence has been discussed in previous reports (25, 26). The present results are consistent with these previous studies in showing that rDNA is more highly methylated in older mice (Fig. 6). DNA methylation is known to repress transcriptional activity (25). Indeed, the ratio of 45S to 28S transcripts decreased in the old BALB/cA mice. The underlying reason for the age-dependent increase in methylation has not been elucidated. However, repetitive noncoding elements, such as retrotransposons, are known targets for DNA methylation enzymes (35). In addition, rDNA is subject to DNA damage and has a high GC content, factors which are known to be related to age-dependent methylation (43, 44). Hence, a similar mechanism may recognize the repetitive rDNA as a target for methylation. Moreover, in terms of the relationship between reduced rDNA transcription and increased copy number in older cells, one possible explanation is that cells can compensate for decreased production of rRNA by elevated copy numbers of rDNA to enable them to survive. As a result, the rDNA copy number in the old mice is greater than that in the young mice.

In this study, although we identified three old-mouse-specific mutations, it is not known whether they occurred during the aging process. Moreover, it is not known whether the rDNA copies with the mutation are actually transcribed or not. Even if these mutated genes are transcribed, the amount of corresponding transcript could be too low to be phenotypically relevant. Thus, other mutations may be related to senescence in the mice. Nonetheless, we found that two equivalent mutations in the budding yeast permitted normal cell growth, but one of the mutations (A3295G) apparently shortened the chronological life span. These findings indicate that the mutated rDNA, when present as the only source of rRNA, is transcribed and can support the essential functions of the ribosome, but viability during aging is negatively impacted, at least in yeast. Therefore, one could infer that if such harmful mutations accumulate in the rDNA repeats during the course of successive cell divisions, they may cause defects in the ribosomal and cellular functions to induce senescence.

In fact, we anticipated more mutations in the older mice because there are many untranscribed noncanonical rDNA copies (22) and because hematopoietic stem cells are subject to DNA replication stress (23). The untranscribed copies can accumulate mutations, and replication stress increases DNA damage. However, to our surprise, the mutation rate in old mice was similar to that in young mice (Fig. 8 and 9E). One possible reason is that cells have an effective repair system and/or mechanism, such as gene conversion for homogenization, to avoid mutation accumulation (36). Alternatively, there could have been many other mutations that were not detected by our sequence analysis. Because rDNA is a multicopy-number gene, it is difficult to detect mutations present at different positions on each copy. Moreover, because 28S and 18S rDNAs have a higher degree of sequence variation in nature (Fig. 8 and 9E), minor age-specific mutations cannot be detected by our sequence analysis. In this study, we identified three age-specific mutations in the older mice, but these mutations may represent only a small fraction of the total number of such mutations. There are likely to be many other minor mutations that failed to be detected. These minor mutations presumably accumulate in older cells and affect senescence.

In this study, we used two mouse strains, BALB/cA and C57BL/6, for the analyses, and they showed slightly different results. The rDNA copy number in C57BL/6 mice was found to be twice that of BALB/cA mice. Age-dependent alterations in the copy number, transcription, and methylation levels were more prominent in BALB/cA mice. The mutation rate in BALB/cA mice was also higher than that in C57BL/6 mice, and we were able to identify specific mutations only in older mice of the BALB/cA strain. These observations suggest that the BALB/cA strain has a stronger aging phenotype than the C57BL/6 strain. Indeed, of the two mouse strains, BALB/cA is known to be more susceptible to carcinogens. Thus, BALB/cA mice may have a less efficient DNA repair system and a more unstable rDNA region, resulting in an enhanced level of senescence.

## MATERIALS AND METHODS

### Mice.

Young mice (4-week-old BALB/cAJc1 and C57BL/6JJc1 mice) were purchased from CLEA Japan, Inc. (Tokyo, Japan). The old mice (approximately 2-year-old BALB/cAJc1 and C57BL/6JJc1 mice) were from this institute (Institute for Quantitative Biosciences, University of Tokyo, Japan). For the data shown in Fig. 9, both the 8-week-old and 100-week-old C57BL/6JJcl mice were purchased from CLEA Japan, Inc. All experiments were approved by the Animal Experiment Ethics Committees at the Institute for Quantitative Biosciences, University of Tokyo (experiment no. 0210). Experiments were performed in precise accordance with the manual provided by the Life Science Research Ethics and Safety Committee, University of Tokyo.

### Determination of rDNA copy number in single cells.

Bone marrow cells (2 × 10^7^) were isolated and washed three times with 5 ml of phosphate-buffered saline (PBS), and then 1 ml of 0.005% propidium iodide (PI) (P4864; Sigma-Aldrich, St. Louis, MO) was added. Each batch of cells was sorted using a high-speed cell sorter (MoFlo XDR; Beckman Coulter, Brea, CA) into a 96-well plate with qPCR buffer (SYBR Premis Ex Taq [Tli RNase H Plus], catalog no. RR420A; TaKaRa, Tokyo, Japan) supplemented with 0.4 μM primers (Table 3) and 0.24% Nonidet P-40 (45). For qPCR, the plate was applied to a Thermal Cycler Dice Real-Time System II (TP900; TaKaRa) with the following amplification conditions: 98°C for 30 s and then 40 cycles of 95°C for 5 s and 60°C for 30 s. The standard curve was generated by serial dilution of DNA from human retinal pigment epithelial cells (RPE1). The rDNA copy number of RPE1 cells was determined by droplet digital PCR (ddPCR). Briefly, 5 ng of RPE1 DNA was digested with HpaII (NEB, Ipswich, MA), suspended in ddPCR mixture consisting of ddPCR Supermix (no dUTP) (catalog no. 1863023; Bio-Rad, Hercules, CA), target primers/probe (6-carboxyfluorescein [FAM]), and reference primers/probe (VIC fluorescent dye, TaqMan copy number reference assay, human, RNase P, catalog no. 4403326; ThermoFisher, Waltham, MA) and applied to an X200 droplet generator (1864002; Bio-Rad). Each droplet was collected into a 96-well plate (twin.tec semiskirted 96-well plate, 951020362; Eppendorf, Enfield, CT) and detected by PCR using the following conditions: 95°C for 10 min, followed by 40 cycles of 94°C for 30 s, 60°C for 1 min, and then 98°C for 10 min. The signal was detected by a QX200 droplet reader, and the number of positive droplets was calculated using QuantaSoft software (1864003; Bio-Rad). On average an RPE1 cell had 330 rDNA copies.

**TABLE 3:** Primer list

Primer name	Sequence (5′–3′)	Note(s) on use^*a*^
28S_Fw	TGGGTTTTAAGCAGGAGGTG	Fig. 2 and 4, ddPCR
28S_Rv	GTGAATTCTGCTTCACAATG	Fig. 2 and 4
28S_ddPCR_Rv	GACGGTCTAAACCCAGCTCA	ddPCR
probe_28S_Fw	GTTGCCATGGTAATCCTGCT	Fig. 3, 5, and 6
probe_28S_Rv	ACCCAGAAGCAGGTCGTCTA	Fig. 3, 5, and 6
probe_Swi5_Fw	AGGAGTTGATTCTCTCTACC	Fig. 3 and 6
probe_Swi5_Rv	GCATCAAGACAATTGTGGTT	Fig. 3 and 6
45S_Fw	CTCTTAGATCGATGTGGTGCTC	Fig. 4
45S_Rv	GCCCGCTGGCAGAACGAGAAG	Fig. 4
Actb_Fw	GACGGCCAGGTCATCACTATTG	Fig. 4
Actb_Rv	AGTTTCATGGATGCCACAGG	Fig. 4
GAPDH_Fw	ACTCACGGCAAATTCAACGG	Fig. 4
GAPDH_Rv	ATGTTAGTGGGGTCTCGCTC	Fig. 4
B2M_Fw	TACGTAACACAGTTCCACCC	Fig. 4
B2M_Rv	TGCTGAAGGACATATCTGAC	Fig. 4
18S_Seq_Fw	TAAGAGAGGTGTCGGAGAGC	Fig. 7 and 9
18S_Seq_Rv	CTTCTCTCACCTCACTCCAG	Fig. 7 and 9
5.8S_Seq_Fw	GTCGTTCCCGTGTTTTTCCG	Fig. 7 and 9
5.8S_Seq_Rv	GACCGAGAAAGACTGGTGAG	Fig. 7 and 9
28S_Seq_Fw	GGTTGCGTGTGAGTAAGATCCTC	Fig. 7 and 9
28S_Seq_Rv	TACTGGTCGACCTCCGAAGTTG	Fig. 7 and 9
ATP5b_Seq_Fw	GAATAATGGCGGTTCTGTGCAC	Fig. 7 and 9
ATP5b_Seq_Rv	ATGATTCTGCCCAAGGTCTCAG	Fig. 7 and 9
28S_target_probe	6FAM-GCCGACATCGAAGGATCAAAAAGCGAC-BHQ1	ddPCR

a Figure references are given for the relevant experiments.

### Southern blot analysis to detect rDNA.

For Southern blot analysis, 150 ng of mouse DNA was digested with 10 units of BamHI-HF (Fig. 3 and 6), NdeI (Fig. 3 and 6), and SacII (Fig. 6) (all restriction enzymes, NEB) overnight at 37°C. The digested DNA was resolved on a 0.8 to 1.0% agarose gel (in 1× Tris-acetate EDTA [TAE] buffer) and blotted onto a filter. The 28S and SWI5 genes were detected on the same filter using PCR-amplified probes with specific primers (Table 3). For the psoralen cross-linking assay, 2 × 10^7^ bone marrow cells were suspended in 8 ml of Opti-MEM I reduced serum medium (ThermoFisher) and divided into two 6-cm-diameter dishes. A 200-μl solution of psoralen in methanol (200 μg/ml; Sigma-Aldrich) was added to each dish, and only methanol was added to the control dish. Each of the dishes was placed on ice for 5 min and cross-linked using UV-A for 4 min (7 cm apart from the UV light). The UV exposure and psoralen addition cycle were repeated four more times (i.e., five cycles in all). Cells were then scraped and collected by centrifugation (1,800 rpm for 5 min), and the DNA was isolated. A 500-ng aliquot of DNA was digested with 20 units of AflIII (NEB) overnight at 37°C and subjected to Southern blot analysis (1% agarose gel in 1× TAE buffer; 60 V, 18 h). The gel was then exposed to UV (4,000 J/cm^2^) using a UV Stratalinker to reverse the cross-linking (46–48).

### RT-qPCR.

Bone marrow cells (1 × 10^7^) were washed with 5 ml of PBS twice, and the total RNA was isolated using a RNeasy minikit (74104; Qiagen, Hilden, Germany). The solution was subsequently treated with DNase I (79254; Qiagen). The RNA was reverse transcribed to DNA by ReverTra Ace qPCR RT Master Mix (FSQ-201; TOYOBO, Tokyo, Japan), and the DNA solution (0.2 ng) was applied to qPCR using SYBR Premis Ex Taq (Tli RNase H Plus) (RR420A; TaKaRa). For normalization, the housekeeping Actb (actin, beta), GAPDH (glyceraldehyde-3-phosphate dehydrogenase), and B2M (beta-2 microglobulin) genes were also examined. The sequences of the primers (0.4 μM each) are given in Table 3. The PCR conditions were as follows: 40 cycles of 95°C for 5 s and 60°C for 30 s.

### rDNA sequence analysis.

rDNA coding regions (18S, 5.8S, and 28S) were amplified by PCR. The PCR mix included 20 ng of rDNA or 150 ng of ATP5b gene genomic DNA in a 40-μl reaction mixture (0.2 mM deoxynucleoside triphosphates [dNTPs], 1.5 mM MgSO_4_, 0.25 μM primers, 1× PCR buffer for KOD-Plus-Neo, 0.8 U of KOD-Plus-Neo). The sequences of the primers are listed in Table 3. The PCR cycle conditions were as follows: for 18S rRNA, 94°C for 2 min and then 25 cycles of 98°C for 10 s, 60°C for 30 s, and 68°C for 90 sec, followed by 68°C for 15 s; for 5.8S rRNA, 94°C for 2 min and then 25 cycles of 98°C for 10 s, 60°C for 30 s, and 68°C for 30 s, followed by 68°C for 15 s; for 28S rRNA, 94°C for 2 min and then 25 cycles of 98°C for 10 s and 68°C for 3 min, followed by 68°C for 15 s; for ATP5b, 94°C for 2 min and then 25 cycles of 98°C for 10 sand 68°C for 30 s, followed by 68°C for 15 s. The PCR products were purified by a Nucleospin Gel and PCR Clean-up kit (740609-250; TaKaRa). Purified DNA was sonicated using a Covaris M220 instrument (Covaris, Woburn, MA) to 150- to 200-bp fragments. The DNA was purified using a QIAquick PCR purification kit (Qiagen), and the library was generated with an NEB Next Ultra II DNA library prep kit for Illumina (NEB). The quality of the library was checked using an Agilent 2100 bioanalyzer (Agilent High Sensitivity DNA kit). Sequencing was performed by a HiSeq 2500 instrument (Hiseq SR Cluster kit, version 4, and HiSeq SBS kit, version 4 [Illumina]; 50 cycles). The sequence data were mapped on the reference sequence (GenBank accession no. BK000964) using Bowtie 2 (version 2.3.3.1), and the base frequency at each position was calculated to obtain the mutation rate (substitution, insertion, and deletion) on a Galaxy platform (https://usegalaxy.org/).

### Yeast strains.

Yeast strains expressing plasmid-borne rDNA with distinct mutations were constructed by plasmid shuffling. In brief, the rDNA depletion strain NOY986 (*MAT***a** *ade2-1 ura3-1 trp1-1 leu2-3*,*112 his3-11,15 can1-100 rdn*ΔΔ::*hisG*) (49) carrying a high-copy-number rDNA/URA3^+^ plasmid was first transformed with an rDNA/LEU2^+^ plasmid containing a mutation at A3295 (mouse A4614) or at A2131 (mouse A3291) within the 25S region. Strains that had lost the URA3^+^-expressing plasmid were then positively selected on synthetic complete (SC) medium without leucine plates containing 5-fluoroorotic acid (5-FOA).

### Yeast chronological life span analysis.

Yeast cells were streaked on a 2% glucose-yeast extract-peptone (YP) plate from a glycerol stock and incubated at 30°C for 3 days. A single colony was grown at 30°C overnight in 2 ml of SC medium containing 2% glucose, with shaking at 200 rpm. The culture was diluted with fresh 2% glucose-SC medium to an optical density (OD) of 0.1 (OD at 600 nm [OD_600_] units) to give a day 0 culture of 20 ml. Starting at day 3 and every 2 days, a 100-μl aliquot of the culture was removed and diluted with sterile water to prepare a 1:10,000 dilution. The dilution was spread onto a 2% glucose-YP plate and incubated at 30°C for 3 days. The number of CFU was scored and normalized to that of the day 3 culture to establish the survival rate. All experiments were performed in biological triplicates.

### Statistics.

The data in the figures were analyzed for statistical significance by the two-sided Student *t* test.

### Data availability.

All data used for the figures are available in Mendely (https://doi.org/10.17632/wr3kmzjwwk.1). High-throughput sequencing data have been uploaded to NCBI Sequence Read Archive database under accession number PRJNA636244 (https://www.ncbi.nlm.nih.gov/sra).
